# Cervical cancer prevention in reproductive health services: knowledge, attitudes and practices of midwives in Côte d’Ivoire, West Africa

**DOI:** 10.1186/1472-6963-14-165

**Published:** 2014-04-11

**Authors:** Boris K Tchounga, Antoine Jaquet, Patrick A Coffie, Apollinaire Horo, Catherine Sauvaget, Innocent Adoubi, Privat Guie, François Dabis, Annie J Sasco, Didier K Ekouevi

**Affiliations:** 1 Programme PAC-CI, CHU de Treichville, Abidjan, Côte d’Ivoire; 2 Université Bordeaux, ISPED, Centre INSERM U897-Epidémiologie-Bio statistique, Bordeaux F-33000, France; 3 INSERM, ISPED, Centre INSERM U897- Epidémiologie-Biostatistique, Bordeaux F-33000, France; 4 Service des Maladies Infectieuses et Tropicales, Centre Hospitalier Universitaire de Treichville, Abidjan, Côte d’Ivoire; 5 Service de Gynécologie Obstétrique, Centre Hospitalier Universitaire de Yopougon, Abidjan, Côte d’Ivoire; 6 Screening Group, Early Detection and Prevention Section, International Agency for Research on Cancer, Lyon, France; 7 Service de Cancérologie, Centre Hospitalier Universitaire de Treichville, Abidjan, Côte d’Ivoire; 8 Programme National de Lutte contre le Cancer, Abidjan, Côte d’Ivoire; 9 Service de Gynécologie Obstétrique, Centre Hospitalier Universitaire de Treichville, Abidjan, Côte d’Ivoire

**Keywords:** Cervical cancer, Prevention, Screening, HPV vaccine, Midwives, Reproductive health, Africa

## Abstract

**Background:**

Cervical cancer is the most common cancer among women and the leading cause of cancer deaths in women in Côte d’Ivoire. Low resource countries can now prevent this cancer by using HPV vaccine and effective and affordable screening tests. However the implementation of these prevention strategies needs well-trained human resources. Part of the solution could come from midwives by integrating cervical cancer prevention into reproductive health services. The aim of this survey was to assess knowledge, attitudes and practices of midwives towards cervical cancer prevention in Abidjan, Côte d’Ivoire, and to find out factors associated with appropriate knowledge.

**Methods:**

A cross-sectional survey was conducted among midwives in the urban district of Abidjan, using a self-administered questionnaire. Knowledge was assessed by two scores. Factors associated with appropriate knowledge were determined using a logistic regression analysis. Attitudes and practices were described and compare using the Chi^2^ test.

**Results:**

A total of 592 midwives were enrolled, including 24.5% of final-year students. 55.7% of midwives had appropriate knowledge on cervical cancer, and 42.4% of them had appropriate knowledge on cervical cancer prevention strategies. Conferences, courses taken at school of midwifery and special training sessions on cervical cancer (OR = 4.9, 95% CI [1.9 to 12.6], p <0.01) were associated with good knowledge on the management of this disease. Among these midwives, 18.4% had already benefited from a screening test for themselves, 37.7% had already advised screening to patients and 8.4% were able to perform a visual inspection. 50.3% of midwives knew HPV vaccine as a preventive method; among them 70.8% usually recommended it to young girls.

**Conclusion:**

Despite sufficient knowledge about cervical cancer prevention, attitudes and practices of midwives should be improved by organizing capacity building activities. This would ensure the success of integration of cervical cancer prevention into reproductive health services in countries like Côte d’Ivoire.

## Background

Cervical cancer (CC) is the second most common cancer affecting women worldwide, after breast cancer [1]. In 2008, the number of new cases of cervical cancer was estimated at 530,000 and 275,000 deaths were attributed to this disease. Most of these new cases (86%) and deaths (88%) occurred in developing countries mainly in sub-Saharan Africa and South-East Asia [1]. In Côte d’Ivoire, West Africa, CC is the leading cancer in women: in 2008 its incidence and mortality were estimated at 26.9 cases and 19.1 deaths per 100,000 women, respectively [1]. More than 70% of the CC cases are diagnosed at an advanced stage in the country [2].

The carcinogenic role played by high risk Human Papilloma Virus (HPV) in the occurrence of CC is now well documented [3-5]. The frequency and severity of CC, its long natural history marked by the initial appearance of precancerous lesions and their possible evolution to malignancy, as well as the existence of good screening techniques, effective treatment methods, and effective preventive vaccines, make CC a suitable disease for preventive actions [6]. Developed countries significantly reduced the burden of CC by implementing organized national screening programs based on cytology [7-9] and have now made HPV vaccine widely available for young children [10]. Côte d’Ivoire has not succeeded in implementing those actions yet, mainly because of the high cost of HPV vaccine and cytology, as well as the lack of qualified human resources. Recently, the advocacy of Global Alliance for Vaccine and Immunization (GAVI) and the commitment of the pharmaceutical industry to make HPV vaccine available and cheaper for low and middle income countries, led to a substantial reduction of the HPV vaccine cost from $335 to less than $15 for the three recommended doses [11]. Regarding cervical cancer screening program, alternatives to cytology as a screening test are now available and are quite suitable for low-resource settings. One of them is visual inspection screening with acetic acid or Lugol’s iodine, for which recent reports from sub-Saharan Africa showed the feasibility in general population as well as in high-risk populations such as HIV-positive women [12-14]. In fact, some studies showed that this technique has a diagnostic accuracy close to cytology and could be performed by paramedical staff [15,16].

The World Health Organization (WHO) currently recommends the involvement of doctors, nurses, midwives and all available health agents in cervical cancer prevention [17]. However, the first step of this strategy is to ensure that health agents have appropriate knowledge and attitude about cervical cancer so that they could play a sustainable role in the prevention of this disease. In Côte d’Ivoire there are more than 2,500 midwives currently in activity. They are formally trained for three years after high school to become certified midwives. After their graduation, almost all of them are employed by government and become registered midwives who work in government health facilities. Their duties are to manage pregnancy, childbirth and to engage in preventive activities such as the Prevention of Mother to Child Transmission of HIV (PMTCT), family planning as well as vaccination of mothers and children [18,19]. Those midwives, who are in contact daily with mothers and children, could play a major role in the prevention of CC through awareness, screening and vaccination, if these activities were effectively included as part of their scope of work. Although WHO recommendations related to CC prevention are currently part of their training program and that some of them have already been involved in prevention of CC through pilot programs, there is no information available on their general knowledge, attitudes and practices toward CC and its prevention.

The aim of this study was to document the knowledge, attitudes and practices of midwives about CC prevention in order to allow integration of prevention activities for this disease as part of their duties in Côte d’Ivoire.

## Method

### Study design and setting

A cross-sectional survey was conducted from April 26 to June 29, 2012 among midwives working in government health facilities in the urban area of Abidjan in Côte d’Ivoire.

### Selected sites

Like many developing countries, Côte d’Ivoire has a pyramidal health system with three levels, each corresponding to a specific type of health facility. From top to bottom, the tertiary level corresponds to University Hospitals (UH); the secondary level corresponds to General Hospitals (GH) and the primary level corresponds to Primary Health Centers (PHC). Midwives who work in these health facilities are trained in schools of midwifery during three years. During the study period, there were three University Hospitals, 11 General Hospitals (including three military hospitals) and 63 Primary Health Centers distributed in the 11 health districts of the urban area of Abidjan [20]. This study was conducted in the corresponding wards of the three University Hospitals, and eight of the 11 General Hospitals located in the economic capital of Côte d’Ivoire. The three military General Hospitals were not visited, due to defense department procedures. For the PHC, in each health district we selected the two centers which had the largest number of midwives. We also selected the only one school of midwifery in Abidjan and conducted the study among students in their final year. This last group was selected given the fact that in Côte d’Ivoire, students in final year of school of midwifery attend government hospitals for their internship and during this period they are involved in all clinical activities including disease prevention. After their graduation, they can spend one or two years before being officially registered by the government, while waiting, they have a special authorization to perform as trainees in government hospitals where they have the same duties than registered midwives.

### Participants’ selection and enrolment procedure

This study was conducted with an exhaustive approach. The aim was to reach all the midwives working in the government health facilities in the urban area of Abidjan. Each selected site was visited by an investigator during five days from Monday to Friday, from 8 am to 4 pm. During this period all the midwives present on site were approached individually and invited to participate in the study. The investigator explained the purpose of the study and after giving their oral consent, the midwives were included in the study and received a self administered questionnaire.

### Data collection

By the time of the study there was, to our knowledge, no validated questionnaire or score assessing knowledge, attitudes and practices of health professionals regarding cervical cancer. For this study, we developed a survey form and a scoring system which are available on line (http://mereva.isped.u-bordeaux2.fr). These tools were constructed based on a consensus from a multidisciplinary workgroup, consisting of gynecologists, oncologists, epidemiologists and senior midwives. In a pilot study, the survey form was tested on a small sample of 50 senior midwives; all of them being unit supervisors. This allowed us to determine the time required to complete a form and to better rewrite complex and confusing questions. Three midwives specialized in public health were recruited and trained to conduct this survey as field investigators. Knowledge, attitudes and practices of midwives were collected through survey instrument, under the supervision of the investigators. The survey form allowed collecting information on demographic characteristics of midwives, their sources of information on CC, their knowledge, attitudes and practices on CC and its prevention.

### Outcomes

Knowledge of midwives was evaluated using two scores. The first one assessed knowledge on epidemiology, risk factors and symptoms. The second score evaluated knowledge on CC prevention, screening and vaccination. Each score was composed of 15 questions, each worth one point. Questions were grouped into three categories and each of them addressed specific issues as described in Table 1. For each question, when the correct answer was provided, the mark assigned to the question was added to score. The maximum value of each score was 15 points, equivalent to 100%.

**Table 1:** Construction of scores for the assessment of knowledge on cervical cancer among midwives in Abidjan, Côte d’Ivoire, 2012

**Items studied**	**Topics**	**Number of questions**	**Theme of questions**	**Topic value**	**Total score**	**Scale of analysis**
			Incidence			
	Epidemiology	5	Mortality	Min = 0 Max = 5		
			Age			
Score of knowledge on cervical cancer disease			HPV		15	0- 100%
	Risk factors	5	Sexuality	Min = 0 Max = 5		
			Multiparity			
	Symptoms	5	Bleedings	Min = 0 Max = 5		
			Pains			
			Vaccine			
	Prevention method	5	Screening	Min = 0 Max = 5		
			Treatment of low			
Score of knowledge on cervical cancer prevention			grade lesions		15	0- 100%
	Screening	5	Type of test	Min = 0 Max = 5		
			VIA			
	Vaccination	5	Availability	Min = 0 Max = 5		
			Target			

VIA: Visual Inspection with Acetic Acid or Lugol’s Iodine.

### Statistical analysis

We created two variables: “Knowledge of CC” corresponding to the first score and “Knowledge of prevention” corresponding to the second one. Each variable was dichotomized into “Appropriate” when the score was >70% and “Inappropriate” when the score was < = 70%. Both were the dependent variables, while demographic characteristics and sources of information were independent variables.

Homogeneity Chi square test and Fisher’s exact test were used to compare proportions, while Kruskal-Walli’s test was used to compare medians in different groups of midwives considered. Determinants of knowledge were investigated by multivariate logistic regression analysis using a step-down method. Odds ratios were adjusted for the type of health facilities, professional experience, professional status, and the different sources of information on cervical cancer. P values <0.05 were considered statistically significant. Statistical analyses were done with Epiinfo software (Epi Info™ version 3.5.3 CDC Atlanta Georgia).

### Ethical aspects

This study was part of the IeDEA study Protocol in Côte d’Ivoire that had already been approved by the National Ethics Committee of the Ministry of Public Health of Côte d’Ivoire. The Ministry of Public Health gave a specific approval for this study. The regional health coordinator and the health district coordinators have authorized the study as well as the coordinators of each health facility in which the survey was conducted. The oral consent of respondents was obtained after explaining the objectives of the study and ensuring the confidentiality.

## Results

### Study population

A total of 695 midwives were present in the selected sites during the study period. Among them, 51 (7.3%) refused to participate and 52 (7.5%) returned incomplete survey forms (answered to less than half of the questions), mainly because they had been called for duty. Overall 592 midwives (85.2%) accepted to participate in the study and correctly completed their survey forms. Their distribution by type of health facility was the following: 125 (21.1%) working in UHs, 134 (22.6%) in GHs, 194 (32.8%) in PHCs and 139 (23.5%) were midwives students in their final year at the school of midwifery of Abidjan (SoM).

### Demographic characteristics and information sources

The median age of the interviewees was 37 years; inter quartile range (IQR) [32–45]. Among certified midwives, the median duration of professional experience was 11 years, IQR [4–22] and 421 (71.1%) of them were registered midwives (regularly working in a government health facility). The main information sources of midwives on CC was colleagues (70.0%) followed by courses taken at school of midwifery (60.8%); 18% of midwives had attended conferences on CC but 6.0% only had taken part to special training sessions on CC prevention.

### Knowledge on cervical cancer disease

Overall, 359 (55.7%) of midwives had appropriate knowledge (score >70%) about epidemiology of CC, risk factors and symptoms. There was no statistical difference in knowledge between the three professional locations (p = 0.94). Midwifery students had significantly less knowledge on CC disease than certified midwives (p = 0.02; Table 2). More than 99% of midwives correctly identified CC as the most common and most deadly cancer of women in Côte d’Ivoire. The three main risk factors reported were: multiple sexual partners (85.5%), early sexual intercourse (80.1%) and HPV infection (69.1%). Midwives working in UH were more aware of the oncogenic role of HPV (78.4%) than those working in GH (63.4%, p < 0.01) and those working in PHC (66.0%, p < 0.01). Post-coital bleeding was correctly identified by 452 midwives (76.4%) as early symptom of CC.

**Table 2:** Knowledge of midwives about cervical cancer disease and its prevention, by category of health facility, Abidjan, Côte d’Ivoire 2012

	**Total**	**UH**	**GH**	**PHC**	**P**	**SoM**	P*
	**N = 592 (%)**	**n = 125 (%)**	**n = 134 (%)**	**n = 194 (%)**		**n = 139 (%)**	
**Score of knowledge of cervical cancer disease**							
Appropriate (>70%)	329 (55.7)	75 (60.0)	80 (59.7)	113 (58.2)	0.94	61 (43.9)	0.02
**Main items of the score of knowledge of cervical cancer disease**							
Most frequent cancer in women^†^	587 (99.2)	123 (98.4)	133 (99.3)	193 (99.5)	0.58	138 (99.3)	0.82
Most deadly cancer in women^†^	584 (98.6)	122 (97.6)	133 (99.3)	192 (99.0)	0.44	137 (98.6)	0.66
HPV infection (risk factor)^†^	409 (69.1)	98 (78.4)	85 (63.4)	128 (66.0)	0.02	98 (70.5)	0.05
Multiparity^†^	317 (53.5)	73 (58.4)	69 (51.5)	113 (58.2)	0.41	62 (44.6)	0.06
Post-coital bleeding (symptoms)^†^	452 (76.4)	96 (76.8)	111 (82.8)	157 (80.9)	0.45	88 (63.3)	<0.01
**Score of knowledge of prevention**							
Appropriate (>70%)	251 (42.4)	66 (52.8)	58 (43.3)	77 (39.7)	0.05	50 (36.0)	0.03
**Main items of the score of knowledge of prevention**							
Cervical cancer is preventable	544 (91.9)	116 (92.8)	125 (93.3)	179 (92.3)	0.94	124 (89.2)	0.60
Screening is a preventive method^†^	421 (71.1)	85 (68.0)	98 (71.3)	150 (77.3)	0.18	88 (63.3)	0.03
Aware of visual inspection as screening method	268 (45.3)	66 (52.8)	56 (41.8)	63 (32.5)	<0.01	83 (59.7)	<0.01
Vaccine is a preventive method^†^	298 (50.3)	70 (56.0)	63 (47.0)	98 (50.5)	0.41	67 (48.2)	0.57
Vaccine administered before sexual debut^†^	259 (43.8)	68 (54.4)	63 (47.0)	80 (41.2)	0.07	48 (34.5)	0.01
Vaccine availability in Côte d’Ivoire	212 (35.8)	51 (40.8)	53 (39.6)	60 (30.9)	0.12	48 (34.5)	0.23

**UH:** University Hospital; **GH:** General Hospital; **PHC:** Primary Health Center; **SoM:** School of Midwifery; **p:** comparison of responses between UH, GH and PHC (student excluded). P*: comparison of the four groups of midwives. ^**†**^Correct answer.

### Knowledge on cervical cancer prevention

Overall, 251 midwives (42.4%) had appropriate knowledge about prevention of CC (score >70%). Midwives working in UH had better knowledge about CC prevention (52.8%) than those working in PHC (39.7%, p < 0.01) and SoM students (36.0%, p < 0.01).

More than 90.0% of midwives knew that CC was preventable and screening was identified as a preventive method by 71.1% of the respondents (Table 2).

In the whole midwives population, Pap smear (88.7%), HPV test (53.9%) and visual inspection methods (49.7%) were correctly identified as screening methods. Less than 10% of the respondents knew the age at which to initiate screening, as well as the periodicity of tests. Only 268 midwives (45.5%) reported knowing exactly what visual inspection of cervix was. Among them, 75.7% were aware that this test gives an immediate result and 65.3% knew that it is achievable by midwives. Midwives working in PHC were less aware of visual inspection (32.5%) than those working in UH (52.8%, p < 0.01), those working in GH (41.8%, p = 0.04) and in SoM students (59.7%, p < 0.01).

Only 298 midwives (50.3%) were aware of HPV vaccine and knew that this vaccine protects against CC. For 43.8% of the respondents, this vaccine should be administered before first sexual intercourse and 38.5% of them knew that it was already available in Côte d’Ivoire.

### Attitude and practice of midwives toward cervical cancer

In this sample, 112 midwives (18.9%) had completed CC screening, mainly by Pap smear (13.5%) and visual inspection (4.9%) (Table 3). About a third of midwives (37.7%) routinely advised screening to their patients. In addition, midwives who had already been screened more often proposed screening to their patients (OR = 5.9; 95% Confidence Interval (CI) [3.7-9.2], p <0.01).

**Table 3:** Attitudes, practices and opinion of midwives toward cervical cancer prevention by category of health facility, Abidjan, Côte d’Ivoire, 2012

	**Total**	**UH**	**GH**	**PHC**	**P**	**SoM**	P*
	**N = 592 (%)**	**n = 125 (%)**	**n = 134 (%)**	**n = 194 (%)**		**n = 139 (%)**	
**Midwives screened**	112 (18.9)	39 (31.2)	26 (19.4)	32 (16.5)	<0.01	15 (10.8)	<0.01
**Type of test (N = 112)**					NA		NA
Pap smear	80 (13.5)	29 (74.4)	19 (73.1)	27 (84.4)		5 (33.3)	
VIA	29 (4.9)	8 (20.5)	7 (26.9)	5 (15.6)		9 (60.0)	
Ignore it**	3 (2.7)	2 (5.1)	0 (0.0)	0 (0.0)		1 (6.7)	
**Practice of screening**							
Propose screening to women	223 (37.7)	57 (45.6)	62 (46.3)	53 (27.3)	<0.01	51 (36.7)	<0.01
Had already performed visual inspection	50 (8.4)	12 (9.6)	16 (11.9)	11 (5.7)	0.12	11 (7.9)	0.22
**Opinion on the HPV vaccine (N = 298)**							
Protect against cervical cancer	224 (75.2)	64 (80.0)	47 (79.7)	75 (81.5)	0.01	38 (56.7)	<0.01
Available in Côte d’Ivoire	158 (53.0)	42 (52.5)	39 (66.1)	47 (51.1)	0.12	30 (44.8)	0.11
Dangerous for health	10 (3.4)	2 (2.5)	3 (5.1)	4 (4.3)	NA	1 (1.5)	NA
Expensive	8 (2.7)	2 (2.5)	2 (3.4)	3 (3.3)	NA	1 (1.5)	NA
Will recommend it	211 (70.8)	59 (73.8)	45 (76.3)	74 (80.4)	0.10	33 (49.3)	<0.01

**UH**: University Hospital; **GH:** General Hospital; **PHC**: Primary Health Center; **SoM:** School of Midwifery; p: comparison of responses between UH, GH and PHC (student excluded). P*: comparison of the four groups of midwives. Ignore it**: respondents report that the test was neither pap smear nor VIA, but were unable to remember the name of the screening test they benefited from. NA: Chi^2^ test Not Applicable.

Among 298 midwives aware of HPV vaccine, 211 (70.8%) would recommend it. HPV vaccine was considered too expensive by only 2.7% of midwives.

### Factors associated with appropriate knowledge on cervical cancer and it prevention

Having a long professional experience was significantly associated with appropriate knowledge on CC disease (aOR = 5.0; 95% CI [2.9-8.7]; p = 0.01) and on CC prevention (aOR = 2.2; 95% CI [1.3-3.6]; p = 0.01) among midwives (Table 4). Courses taken at school of midwifery (aOR = 1.5 95% CI [1.0 - 2.3] p = 0.03); conferences (aOR = 1.78 95% CI [1.1 - 3.0] p = 0.03); and special training sessions on CC (aOR = 4.9 95% CI [1.9 - 12.7] p < 0.01) were significantly associated to appropriate knowledge on CC prevention. Age of midwives (aOR = 1.74; 95% CI [0.74-4.01]; p = 0.19), employment status (aOR = 0.93; 95% CI [0.19-4.50]; p = 0.93) and type of health facility (aOR = 1.30; 95% CI [0.85-2.13]; p = 0.25) were not associated with appropriate knowledge on prevention of CC.

**Table 4:** Factors related to appropriate knowledge on cervical cancer disease and cervical cancer prevention among midwives in Abidjan, Cote d'Ivore: final multivariate analysis

	**Knowledge on cervical cancer disease**				**Knowledge on cervical cancer prevention**			
	**N = 592**	**Adjusted OR**	**[(95%) CI]**	**P**	**N = 592**	**Adjusted OR**	**[(95%) CI]**	**P**
**Attending courses at SoM(ref number N = 232)**								
**Yes**	360	1.56	[1.06 - 2.29]	0.02	360	1.52	[1.04 - 2.24]	0.03
Attending conferences**(ref number N = 482)								
**Yes**					110	1.69	[1.07 - 2.66]	0.02
**Professional experience (years)**								
**<1**	171	-	-	-	171	-	-	-
**1-10**	179	1.37	[0.86 - 2.16]	0.18	179	1.26	[0.79 – 2.04]	0.32
**11-20**	121	5.00	[2.88 - 8.70]	0.01	121	2.23	[1.33 – 3.73]	0.01
**>20**	121	2.64	[1.57 - 4.44]	0.01	121	1.32	[0.78 – 2.21]	0.28
**Attending training session(ref number N =556)**								
**Yes**	36	3.40	[1.36 - 8.52]	0.01	36	4.44	[1.83 - 10.77]	0.00

**OR:** Odds Ratio adjusted for “Attending courses at SoM”, “Attending conferences”, “Professional experience”, “Attending training session”**. CI**: Confidence Interval 95% **SoM**: School of Midwifery **UH**: University Hospital; **GH:** General Hospital; **PHC**: Primary Health Center.

Attending conferences**: variable not considered in the final model of knowledge of cervical cancer disease because in univariate analysis its P Value was not significant (p = 0.96).

## Discussion

This study reveals that more than half of the midwives surveyed in Abidjan had appropriate knowledge on CC disease and more than 40% had appropriate knowledge on CC prevention, specifically by screening and vaccination. Factors associated with appropriate knowledge were professional experience, courses on CC taken at school of midwifery, participation in conferences and training workshops on CC. Moreover, the majority of midwives were aware of screening and more than one third of them had regularly recommended it to their patients. Finally, more than half of midwives knew the existence of HPV vaccine.

The proportion of midwives with appropriate knowledge regarding epidemiology, risk factors and symptoms of CC is higher in this study than in other reports. A previous survey conducted among nurses in Tanzania, reported a proportion of adequate knowledge on cervical cancer of < 40% [21]. This difference could be explained by our study population exclusively made up of midwives who received specific courses on gynecologic cancers at the school of midwifery and who usually manage diseases of the reproductive system.

Studies conducted among wider sample of nurses and other health workers on knowledge of prevention of CC were reported in Pakistan (screening 54%; vaccine 9%), in India and Tanzania (18% and 22% for vaccine, respectively) [21-23]. The relatively high level of knowledge on prevention of CC in our study could be explained by the fact that midwives work closely to gynecologists who perform screening and give advice to patients. Therefore, they are more likely to be well informed on CC prevention than other health professionals. This argument is reinforced by the fact that the most frequently reported information source was colleagues (70%) including gynecologists and midwives with more experience.

On the other hand, the fact that one-third of midwives did not have appropriate knowledge on CC prevention reveals unequal access to information. Indeed, in our study sample, only 18.0% of midwives attended conferences and less than 6.0% took part in seminars or training sessions on CC. Although these proportions are higher than in Tanzania (8.0% and 2.9%, respectively), they are low considering the fact that participation to such activities was associated to good knowledge in our study [21]. This association was not investigated in other reports. Yet, it is important to notice that age, employment status and type of health facility that had been reported in other studies as being associated with good knowledge, remained not significant in our study when adjusted on information sources. Conferences and training sessions usually take place in university hospitals; midwives of these health facilities are then more likely to participate in such activities and may exhibit better knowledge than others.

In our study, 18.4% of midwives had already been screened for CC, and 37.7% often proposed screening to their patients. The low proportion of midwives screened was already reported in previous studies conducted in Nigeria and Uganda, where the authors found respectively 20.5% among 200 nurses and 19.0% among 310 nurses [24,25]. Mutyaba *et al.* in Uganda reported that this low proportion could be the result of negligence and/or the feeling of invulnerability induced by the fact of being a health professional [24]. This highlights the importance of encouraging midwives to get tested, since this study also revealed that midwives screened were more likely to propose screening to their patients.

We found out that only 8.4% of midwives had already conducted a visual inspection. This low proportion is consistent with the fact that visual inspection is not yet routinely used in the country and reflects the absence of national guidelines on the practice of CC screening. Of note this guideline is currently being written for Côte d’Ivoire. More than 50% of midwives were aware of the existence of an HPV vaccine and among them 71% would recommend it. McGarey *et al.* reported similar findings in Cameroon [26].

Our observations highlight the importance of relying on midwives to sensitize women to CC prevention. More than half of the female populations in the country are in their childbearing age. They attend reproductive health facilities where they are offered routine care and prevention services by midwives. Introducing CC prevention into reproductive health services managed by midwives could reinforce the awareness of the disease on the target population and could strengthen the national cancer control program. Since more than half of midwives in Abidjan already have appropriate knowledge on CC prevention, they could provide correct information and convince women to get screened and to have their children vaccinated.

The relatively high proportion of midwives lacking appropriate knowledge is mainly due to the difficulty of access to information. Thus, expanding access to information by organizing seminars and training sessions specifically designed for paramedical staff in all types of health facilities could significantly increase the proportion of midwives with adequate knowledge on CC prevention.

The low practice of CC primary (vaccine) and secondary prevention (screening for cervical precancerous lesions), and the inappropriate attitudes found in this sample, demonstrate once again the lack of comprehensive policy for CC prevention and insufficient communication about the HPV vaccine that has been available in the pharmacies of Abidjan since 2009. Inappropriate attitudes and practices of midwives argue in favor of the adoption of a comprehensive policy for CC prevention and also emphasize the need to adopt national guidelines for screening and vaccination.

This study was the first specifically conducted among a large sample of midwives to assess their knowledge, attitudes and practices toward CC in West Africa. Midwives from all categories of the health care system were represented. Moreover, the procedure of data collection allowed us to retrieve all the survey forms and to limit missing data. Thus, the results of this study accurately reflect the current knowledge of midwives in Abidjan.

One of the limitations of this study is the use of a questionnaire and a scoring approach not validated to assess knowledge of midwives as well as the 70% threshold chosen with reference to national academic grading systems. The study was limited to urban areas where most health facilities have a reproductive health unit. Therefore, results and recommendations may not be applicable to rural areas where nearly 40% of the populations of Côte d’Ivoire live [27] and which are poorly equipped in material and human resources. Despite its limitations, this study covered almost all midwives in the urban area of Abidjan and public health recommendations arising from these results could benefit more than 5 million inhabitants in Côte d’Ivoire.

## Conclusion

This survey showed that nearly half midwives in Abidjan had appropriate knowledge to carry on CC prevention. As their practice is limited and attitude remain suboptimal, they should benefit from capacity building activities as part of a comprehensive strategy leading to the integration of CC prevention in reproductive health services. This could be an interesting starting point for many low-resource countries that have not yet implemented a CC prevention program.

## Abbreviations

CI: Confidence interval; GH: General Hospital; HIV: Human immunodeficiency virus; HPV: Human papilloma virus; IQR: Inter quartile range; OR: Odds ratio; PHC: Primary Health Center; PMTCT: Prevention of mother to child transmission of HIV; SoM: School of midwifery; UH: University Hospital; WHO: World Health Organization.

## Competing interests

The authors declare that they have no competing interests.

## Authors’ contributions

BT, AJ, PAC, DKE designed the study. The survey form and score calculation model was developed and validated by BT, AJ, PAC, DKE, CS, AH, AJS. Data collection was made and supervised by BT, PAC, IA, PG, AH. Analysis and interpretation of data was performed by BT, PAC, AJ, and DKE. The manuscript was drafted by BT, and critical revision for important intellectual content was provided by AJ, PAC, DKE, CS, AH, FD. All authors read and commented on the original manuscript and all agreed on the version finalized by BT for submission.

## Authors’ information

For the IeDEA West Africa collaboration.

## Pre-publication history

The pre-publication history for this paper can be accessed here:

http://www.biomedcentral.com/1472-6963/14/165/prepub
